# High-Reflectance Low-Emissivity Multilayer Wallpaper for Envelope Cooling and Energy Saving of Tropical Buildings

**DOI:** 10.3390/nano16201325

**Published:** 2026-10-20

**Authors:** Jinjing Liu, Yangyang Ge, Zhenghui Shen, Shenghui Han, Weixin Luo, Ruqiang Zou

**Affiliations:** 1 State Key Laboratory of Advanced Waterproof Materials, School of Materials Science and Engineering, Peking University, Beijing 100871, China; 2401120106@stu.pku.edu.cn (J.L.);; 2 State Key Laboratory of Advanced Waterproof Materials, Beijing Oriental Yuhong Waterproof Technology Co., Ltd., Beijing 101300, China; geyy@yuhong.com.cn (Y.G.);

**Keywords:** reflective wallpaper, solar reflectance, low emissivity, radiant barrier, building energy efficiency, tropical climate, multilayer polymer composite

## Abstract

Buildings account for a large share of global energy use, and tropical cooling loads are intensified by solar radiation and humidity. This study characterizes a tape-applied four-layer wallpaper (PET grid fabric/PVC/commercial aluminized film/PE) on one model house per finish in Hainan, China. Laboratory solar reflectance is approximately 0.77 and comparative 8–13 µm emissivity is 0.32–0.38, indicating a solar reflector plus radiant barrier rather than a high-emittance sky-window cooler. In May 2022, EE-1 reduced exterior-roof and indoor-air temperatures by up to 8 °C and 4 °C relative to a contemporaneous uncoated house. Treatments were not randomized, and no pre-treatment baseline exists, so the contrasts are descriptive. Electricity use was 10% and 22% lower at 23 and 20 °C; the 2% contrast at 26 °C is not primary evidence. After 3–4 months, EE-1 delaminated. Retrieved EE-1 retained solar reflectance of 0.769, while transverse elongation fell to 58% of the unexposed value. EE-3/EE-4 improved short-term appearance only. Cooling and multi-month durability were not demonstrated for one formulation.

## 1. Introduction

The building sector is responsible for approximately 20–40% of global final energy consumption [1,2], of which heating, ventilation and air-conditioning (HVAC) systems typically constitute the dominant share. In tropical and subtropical climates, the cooling demand is especially severe: annual solar radiation can exceed 130 kcal cm^−2^ yr^−1^, ambient temperatures frequently rise above 30 °C, and relative humidity often remains between 70% and 94% [3]. Conventional thermal insulation primarily impedes conduction but leaves exterior surfaces free to absorb solar radiation that is then conducted inward; high humidity can further degrade effective resistance and raise condensation risk [4,5].

Two related envelope strategies should be distinguished. Passive daytime radiative cooling (PDRC) combines high solar reflectance in the solar spectrum (wavelength λ = 0.3–2.5 µm) with high emittance in the atmospheric transparency window (wavelength λ = 8–13 µm) so that heat can be radiated to the cold sky [6,7,8,9,10,11]. Cool roofs and high-emittance coatings have lowered surface temperatures by 10–20 °C and cut cooling energy by 10–30% in field studies [4,12,13]. Most of these products are rigid and intended for horizontal roofs, which limits wall and interior retrofit [5].

A second strategy is the radiant barrier or low-emissivity (low-e) finish: a solar-reflective, infrared-reflective surface that reduces absorbed solar load and long-wave exchange with hot adjacent surfaces, the ground or indoor surroundings. Short-wave (solar) radiation refers here to wavelength λ = 0.3–2.5 µm, near-infrared (NIR) to λ ≈ 0.78–2.5 µm, mid-infrared to the Fourier-transform infrared (FTIR) scan range λ ≈ 2.5–25 µm, and long-wave to thermal infrared radiation at approximately λ = 3–50 µm. Mid-infrared emissivity of a radiant barrier is deliberately low, which is the opposite optimization from an ideal PDRC emitter. High solar reflectance is still required; low emittance alone does not reject the daytime solar load. Flexible wallpaper that embeds an aluminized film in protective polymers is a dual-use (exterior and interior) format for this function.

Flexible films have already shown promise in building thermal management. Hierarchical porous cellulose-acetate films reduced surface temperature by 7–17 °C under 700–820 W m^−2^ [14], and thermochromic inorganic wallpapers achieved adaptive cooling of up to 5 °C with solar reflectance of ~0.94 and window emissivity of ~0.96 [15]. Coupled laboratory characterization and full-scale tropical tests of a low-e wallpaper remain scarce, especially under South China Sea monsoon humidity and radiation.

**Figure 1:**
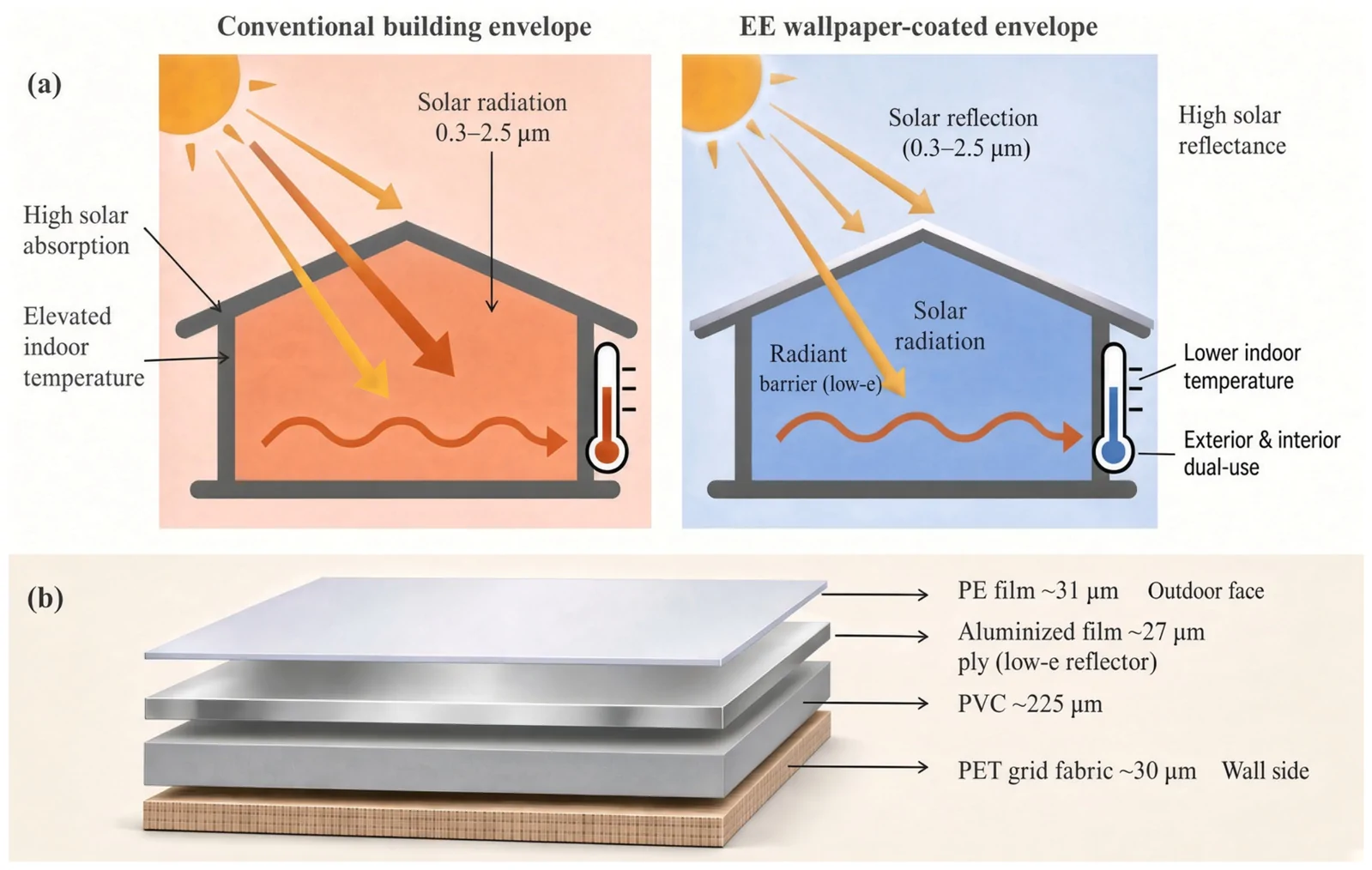
Schematic of the four-layer high-reflectance, low-emissivity wallpaper. (**a**) Conventional envelope and EE-coated envelope, showing solar reflection (λ = 0.3–2.5 µm) and a low-emissivity radiant barrier. (**b**) Layer stack with PE as the outdoor face and PET fabric toward the wall. The 27 µm dimension is the thickness of the aluminized-film ply. Arrows indicate incident solar radiation, reflected solar radiation and the low-emissivity radiant-barrier path.

**Figure 2:**
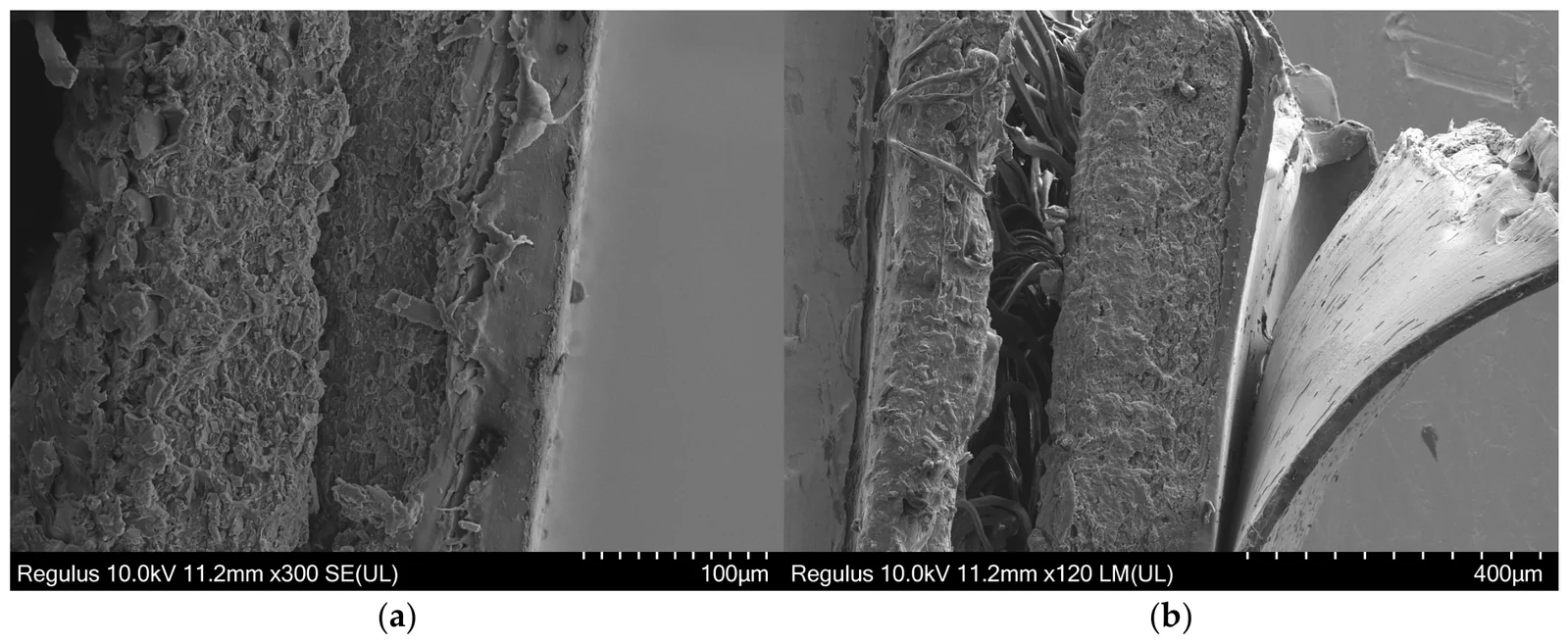
Scanning electron micrographs of EE-1. (**a**) Fracture cross-section of an as-produced sheet (10.0 kV, ×300; scale bar 100 µm). A particle-filled ply consistent with the PVC core lies against a thinner compact polymer skin. (**b**) Coupon retrieved after outdoor exposure in Hainan, March–July 2022 (10.0 kV, ×120; scale bar 400 µm). The laminate is opened; coiled filaments in the gap are assigned to the PET grid fabric, and a continuous film has separated from the filled core.

Here, we report a tape-applied four-layer wallpaper (Yichuang EE-1, EE-2 and later durability-oriented EE-3/EE-4): PET grid fabric (~30 µm)/PVC (~225 µm)/commercial aluminized film (~27 µm)/PE (~31 µm). The configuration is distinguished from rigid roof coatings and from bare-foil radiant barriers by the flexible laminate and the tape application on walls and roofs, not by a new nanostructure. The metal interlayer reflects solar and near-infrared radiation; the polymer skins raise 8–13 µm emissivity from the very low values of bare aluminum to 0.32–0.38, still far below commercial high-e references (Figure 1). The stack is designed to reject solar heat and limit radiative exchange with warm surroundings, not to maximize sky-window emission. Micrometre-scale SEM of as-produced and field-exposed EE-1 is reported in Figure 2. The metallic coating on the aluminized ply is not resolved at the magnifications used, and energy-dispersive maps were not collected.

Relative to dielectric-on-metal photonic coolers [6], the wallpaper avoids precious-metal specular finishes, uses a simpler process and can be taped onto walls, roofs and interiors. The goal is a candidate tropical retrofit finish rather than sub-ambient cooling under ideal sky-facing conditions.

## 2. Materials and Methods

### 2.1. Material Description and Structure

**Figure 3:**
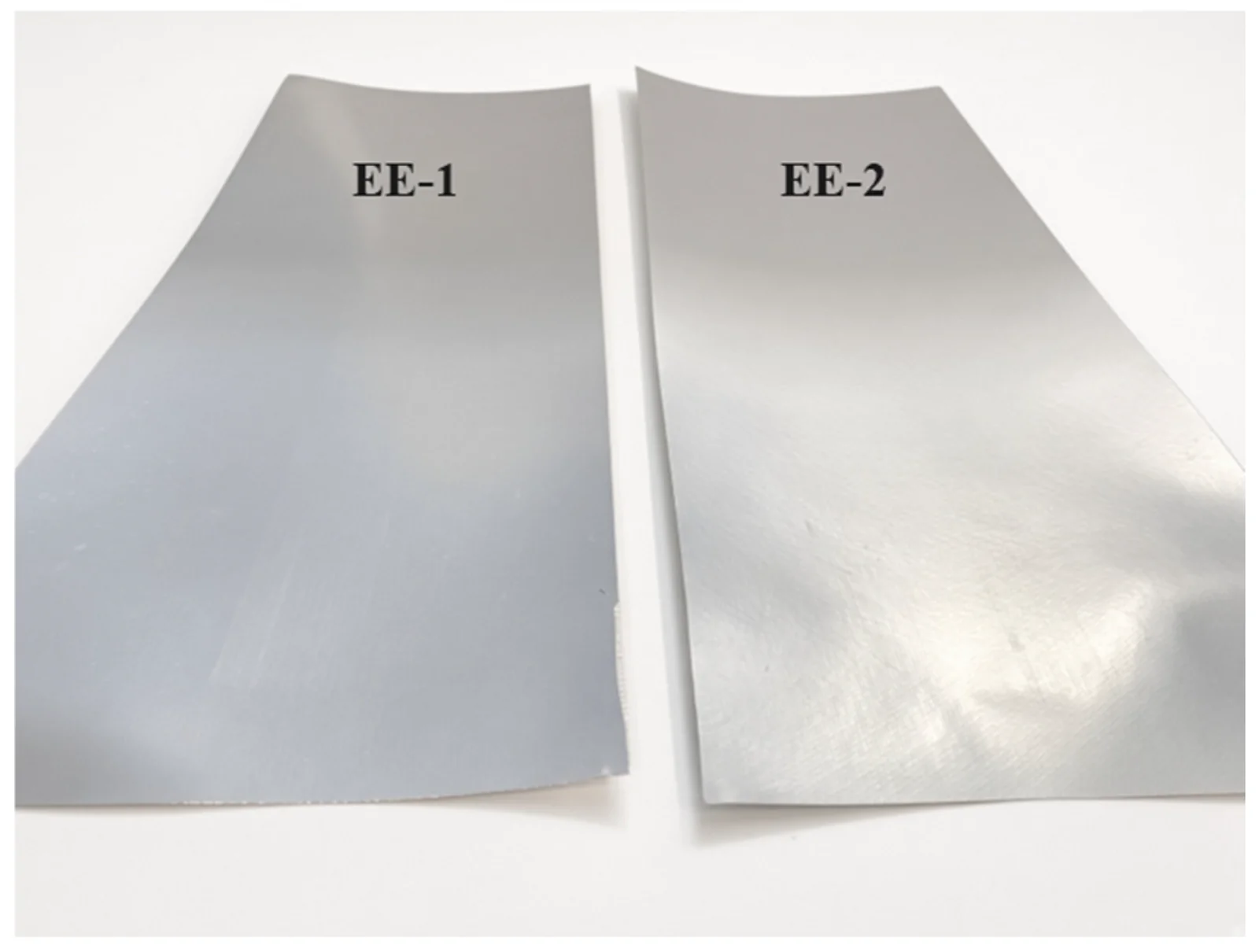
Application of the EE multilayer wallpaper on exterior walls and roofs.

A wallpaper intended for both exterior and interior retrofit must be flexible and optically functional, rather than a rigid roof-only coating. The present design therefore sandwiches a continuous aluminized film between polymer layers: PET grid fabric (~30 µm)/PVC sheet (~225 µm)/aluminized film (~27 µm)/PE film (~31 µm). The metal interlayer is the primary reflector; the polymers protect it, add a moisture barrier and allow tape application. EE-1 and EE-2 were the primary optical, mechanical and instrumented-house articles (Figure 3). EE-3 and EE-4 are later durability-oriented variants that retain the aluminized reflector but change adhesion promoters and the PE formulation; they were not the May–June instrumented finishes except for a short August check of EE-3 on house 5#. 3M VHB GPL110GF waterproof double-sided tape (3M Company, Saint Paul, MN, USA) over two-layer putty. Commercial DAW reflective coating and DG wallpaper were colour-matched to the EE series. House assignment on site was fixed and not randomized: house 1# uncoated control; house 2# DAW; house 3# DG; house 4# EE-1; house 5# EE-2 (exterior finish of house 5# later replaced by EE-3 after EE-2 failed). A pre-treatment baseline in which all five houses ran uncoated under the same weather is not available.

Optical scatter in a multilayer film is sensitive to thickness variation, so layer uniformity is a manufacturing requirement rather than a cosmetic detail. Thickness mapping indicated layer uniformity within ±3 µm, consistent with the later low scatter in reflectance. Compared with single-layer coatings, the multilayer stack is intended to combine flexibility with an improved moisture barrier under tropical conditions.

### 2.2. Laboratory Characterization

Mechanical properties were measured against PVC-sheet methods: width and thickness (GB/T 6672, GB/T 6673 [16,17]), tensile strength and elongation (GB/T 18173.1-2012 [18]), interlayer peel (GB/T 8808-1988 [19]), heat ageing at 70 °C for 720 h (GB/T 18244-2000 [20]), artificial weathering for 720 h (GB/T 16259-2008 [21]), low-temperature bending (GB/T 18426 [22]) and wash resistance for 2000 cycles (GB/T 9755-2014 [23]). Solar and NIR reflectance (wavelength λ = 0.3–2.5 µm and λ ≈ 0.78–2.5 µm) were obtained with an integrating-sphere spectrophotometer following GB/T 25261-2018 [24] (building reflective thermal-insulation coatings). Directional reflectance from 2.5 to 25 µm was measured by FTIR. A calibrated blackbody was not available in that campaign, so 8–13 µm emissivity is a comparative estimate ε = 1 − ρ after averaging over wavelength λ = 8–13 µm, not an independently calibrated hemispherical emittance. Replicate reflectance on EE-1/EE-2 varied by ±0.02. EE-3 laboratory sheets were measured in the later campaign report; EE-4 optical values were not tabulated there.

Cross-sections of as-produced EE-1 and of an EE-1 coupon retrieved after the Hainan outdoor exposure (March–July 2022) were imaged on a Regulus field-emission scanning electron microscope (Hitachi High-Technologies, Tokyo, Japan) operated at 10.0 kV (working distance 11.2 mm). The as-produced fracture face was recorded in secondary-electron mode at ×300; the exposed coupon was recorded with the low-magnification detector at ×120. Specimens were examined as fractured surfaces. Layer assignment is morphological and is compared with the nominal stack PET grid fabric (~30 µm)/PVC (~225 µm)/aluminized film (~27 µm)/PE (~31 µm). Energy-dispersive maps were not collected. The metallic coating on the aluminized ply is thinner than the ply itself and is not resolved at these magnifications.

Solar reflectance and hemispherical emissivity of an EE-1 coupon retrieved after the Hainan exposure were measured in a later laboratory check. Solar reflectance followed GB/T 25261-2018. The corresponding UV–Vis–NIR spectrum was recorded on an Agilent Cary 5000 spectrophotometer (Agilent Technologies, Santa Clara, CA, USA) in %R mode from 250 to 2500 nm (the aged reflectance spectrum). Hemispherical emissivity is the laboratory hemispherical value on that coupon; it is not the same measurand as the campaign comparative 8–13 µm estimate ε = 1 − ρ. Post-exposure spectra of other formulations were not included. Only EE-1 coupons can be traced to house 4#, which carried that sheet throughout the campaign; EE-3 was a mid-campaign replacement on house 5#, and a later EE-4 instrument label could not be matched to the archive.

Tensile properties of retrieved EE-1 coupons were measured on Type-2 dumbbells (narrow parallel width 6 mm, gauge length 25 mm, thickness 0.32 mm), separately in the machine direction (MD; *n* = 5) and the transverse direction (TD; *n* = 5). The field-coupon reports were printed under the GB/T 528-1998 [25] heading; the campaign unexposed sheets were tabulated under GB/T 18173.1-2012. The two headings describe the same dumbbell tensile protocol and are treated as one method. Unexposed EE-1 values were 26.5 MPa/81.56% (MD) and 26.92 MPa/74.21% (TD). Interlayer peel after exposure was not remeasured: the retrieved coupons were too small and too few for a valid GB/T 8808-1988 peel series. Mechanical change after exposure is therefore reported from the dumbbell tensile test.

### 2.3. Real-Scale Model-House Field Testing

**Figure 4:**
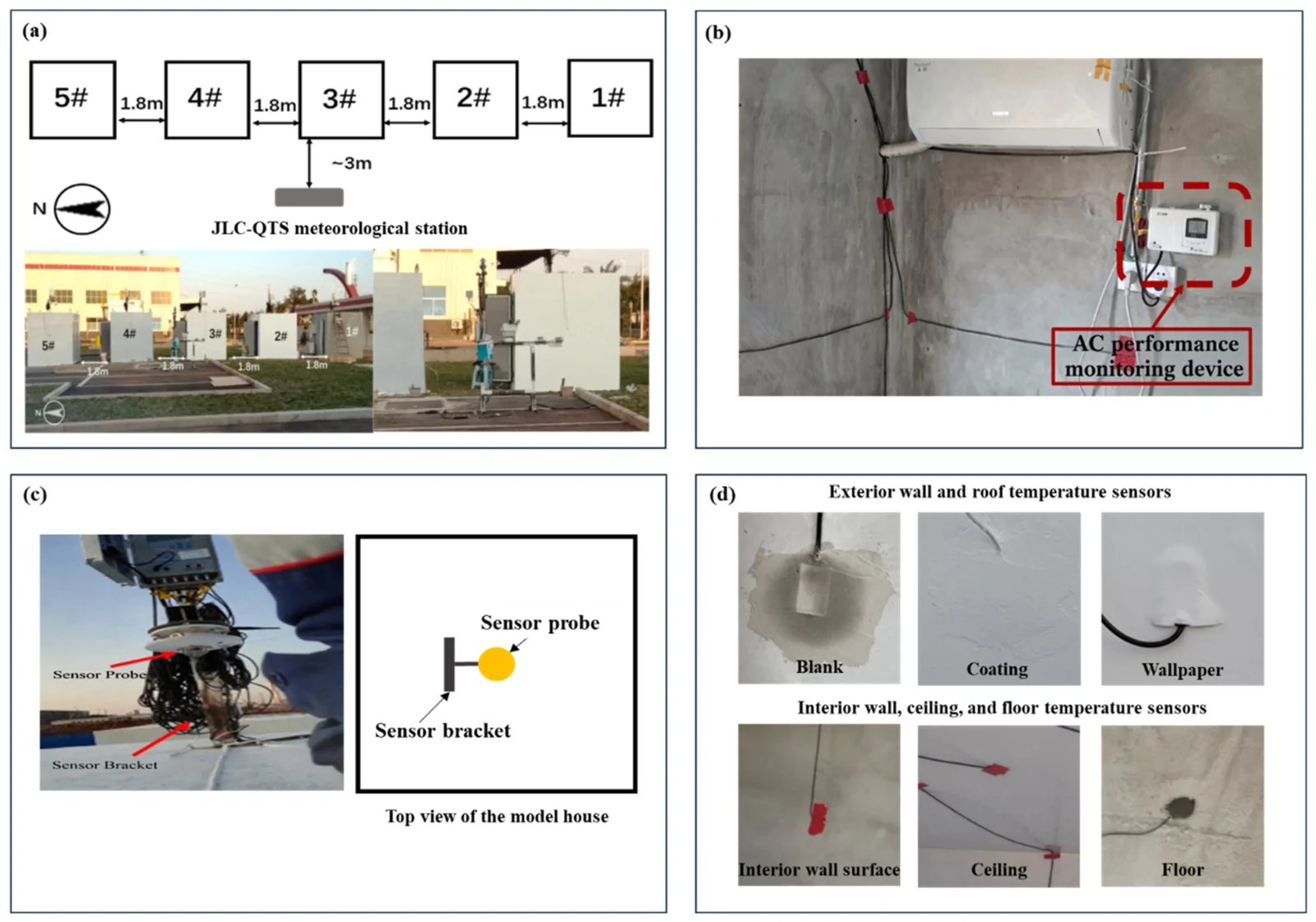
Model-house site and instrumentation. (**a**) Layout of the five cubic houses and the meteorological station. (**b**) Indoor air-conditioners and electricity monitors. (**c**) Roof-mounted broadband radiation sensor. (**d**) Placement of temperature sensors. The labels 1#–5# are the house numbers used in the text.

Laboratory spectra show what the surface can do; they do not show whether that advantage survives a humid, high-radiation monsoon climate on a closed envelope. Five nominally identical cubic houses were therefore built in an unshaded yard at the Oriental Yuhong Yangpu Factory, Danzhou, Hainan Province, China (tropical monsoon climate: annual mean 24.7 °C; solar radiation ~130 kcal cm^−2^ yr^−1^; humidity 26–94%). External dimensions were 2.12 m × 2.12 m × 2.12 m and internal dimensions 1.82 m × 1.82 m × 2.00 m, with 120 mm reinforced-concrete walls finished with putty. Houses faced north–south with 1.8 m spacing. Each house received one finish. Assignment was fixed and not randomized (1# uncoated; 2# DAW; 3# DG; 4# EE-1; 5# EE-2, later EE-3), and no period in which all five houses ran uncoated under the same weather was recorded. Thermal equivalence before coating is therefore assumed from the common construction, not demonstrated. Each house had the same air-conditioner on the upper east wall, a JLC-QTS meteorological station and eleven temperature sensors. Roof-mounted broadband sensors were placed at each roof centre. Air-conditioner electricity was logged with a ZHT-Acmate monitor at 1 min intervals. Pre-installation calibration specified ±0.5 °C for temperature and ±5% for radiation flux. Houses were unoccupied. Neighbour and ground long-wave exchange cannot be excluded at 1.8 m spacing, as shown in Figure 4.

The field campaign was split so that envelope-driven temperature change could be isolated from air-conditioner operation. Construction was completed in early March 2022. Phase 1 was passive (no air-conditioning) monitoring from 14 to 28 March 2022, including the intervals 14–18 March (ambient 23.0–30.2 °C) and 19–22 March (ambient 24.0–37.0 °C), plus 3–5 May 2022 (ambient maxima 26–32 °C). Phase 2 was air-conditioning from 7 May to 24 June 2022 at fixed set-points of 26, 23 and 20 °C (several days to two weeks per condition; ambient maxima 34–37 °C). A later insulated-house check was run on 26–30 August 2022 at 24 °C after interior XPS was added and house 5# received EE-3. Appearance was inspected from March to the end of July 2022 (about four months); EE-3 was inspected through August–November 2022. Wind was 2–7 m s^−1^, humidity 43–94% and broadband radiation 700–1300 W m^−2^ during the main campaigns.

## 3. Results

### 3.1. Laboratory Optical and Mechanical Performance

**Figure 5:**
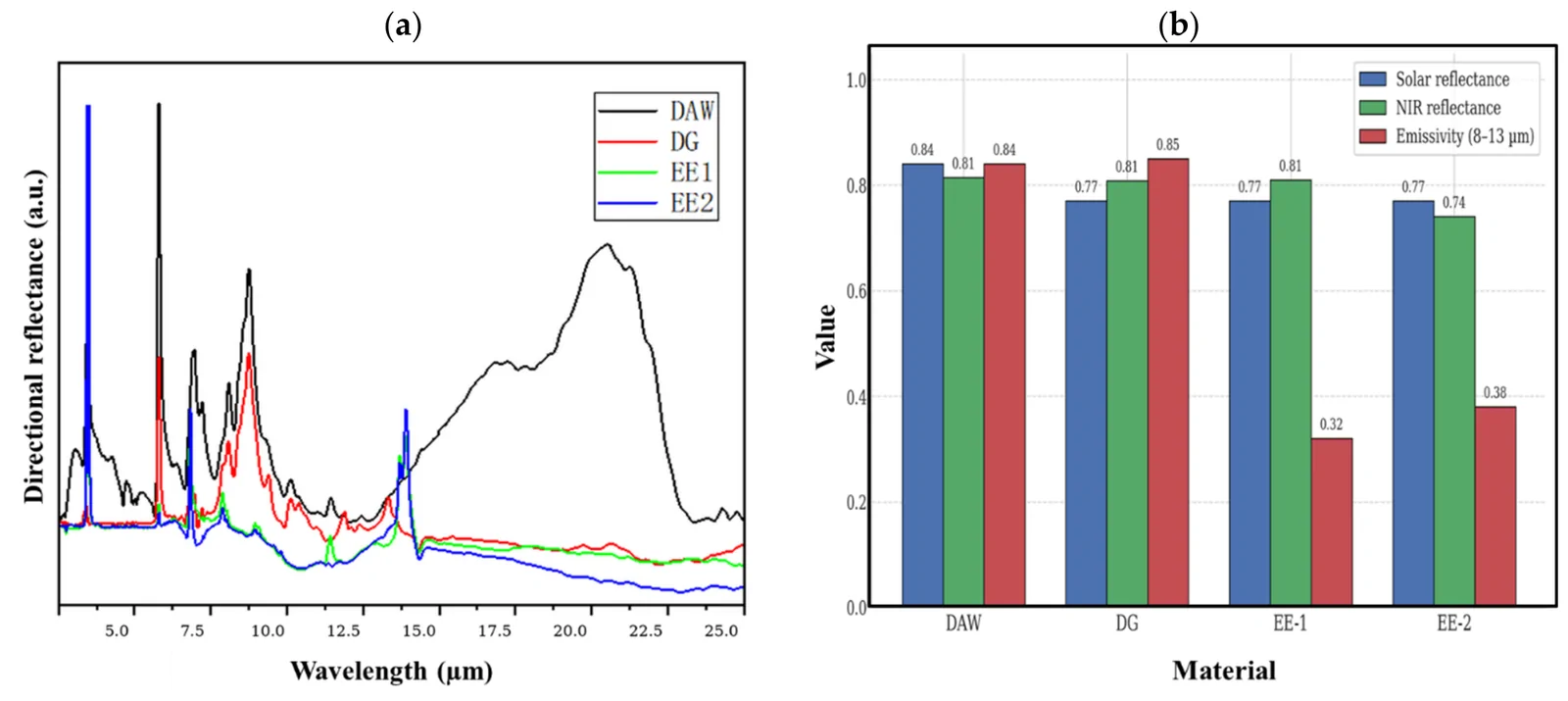
Optical properties of the tested materials. (**a**) Directional FTIR reflectance versus wavelength (λ = 2.5–25 µm), in arbitrary units. (**b**) Solar reflectance (λ = 0.3–2.5 µm), near-infrared reflectance (λ = 0.78–2.5 µm) and comparative emissivity in the 8–13 µm window, obtained as ε = 1 − ρ.

The optical selectivity of the multilayer stack is the physical basis of its cooling function. Solar reflectance was 0.77 for both EE-1 and EE-2 (DAW 0.84; DG 0.77). Near-infrared reflectance was 0.81 (EE-1) and 0.74 (EE-2); the EE-2 NIR value lies 0.01 below the 0.75 threshold quoted from GB/T 25261-2018 in the campaign report. Laboratory sheets for EE-3 give solar reflectance of 0.72 and NIR reflectance of 0.78. Comparative 8–13 µm emissivity was approximately 0.32 (EE-1) and 0.38 (EE-2) versus ~0.84–0.85 for DAW and DG; the latter EE-3 sheet gave 0.52 (Figure 5). Replicate reflectance varied by ±0.02. Because no blackbody reference was used, these emissivities support ranking among the five finishes rather than an absolute sky-window power.

Mechanical integrity is equally essential and already distinguishes the variants. Tensile strength of EE-1, EE-2 and EE-3 was 26.5–30.4 MPa (longitudinal), above the 15 MPa sheet minimum in GB/T 18173.1-2012. Interlayer peel (GB/T 8808-1988) was weak for EE-1 (PE/Al 1.9 N/15 mm; Al/PVC 1.6 N/15 mm) and stronger for EE-2 (4.2 and 5.9 N/15 mm); the PE/Al interface of EE-3 could not be peeled in the same test. After 720 h at 70 °C, tensile-strength retention of EE-1/EE-2 remained at the order of 80–100%. After 720 h of artificial weathering (GB/T 16259-2008), EE-1 discoloured, the PE surface of EE-2 was damaged, and EE-3 showed no obvious laboratory-weathering change. All three variants passed 2000-cycle wash tests (GB/T 9755-2014). These laboratory observations concern as-produced coupons. They explain why EE-1 later delaminated in the field, but they are not post-exposure reflectance or emissivity of weathered sheets.

### 3.2. Outdoor Temperatures Under Tropical Conditions

**Figure 6:**
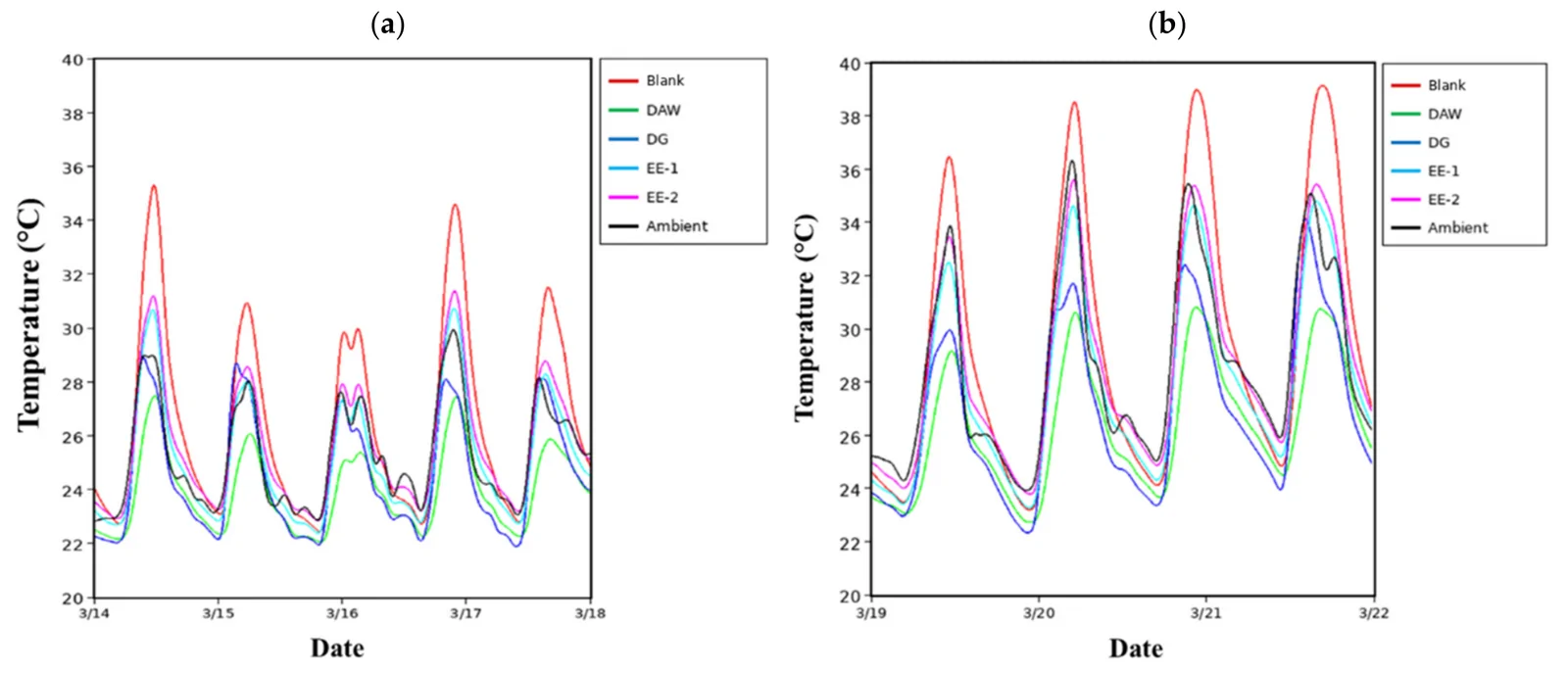
Exterior wall and roof temperatures of the five model houses. (**a**) 14–18 March 2022, ambient air 23.0–30.2 °C. (**b**) 19–22 March 2022, ambient air 24.0–37.0 °C.

To test whether the laboratory optical contrast translates into measurable cooling under tropical monsoon conditions, the five houses were monitored continuously in Hainan. During 14–18 March 2022 (ambient 23.0–30.2 °C), EE-1 and EE-2 exterior walls and roofs were cooler than the blank control at peak solar hours and were also cooler than concurrent ambient air (Figure 6). The advantage increased during 19–22 March (ambient 24.0–37.0 °C). In the 3–5 May 2022 passive tests (ambient peak ~32 °C), EE-1 reduced exterior-roof temperature by 8 °C, interior roof temperature by 5 °C and indoor-air temperature by 4 °C relative to the contemporaneous blank house. EE-1 outperformed EE-2 in all orientations, consistent with its higher NIR reflectance. Differences were largest between 10:00 and 15:00 and persisted across wind speeds of 2–7 m s^−1^ and humidity of 43–94%. Consecutive 1 min samples are not independent replicates; inferential *p*-values are therefore not reported. Because a pre-treatment uncoated baseline of all five houses is unavailable, part of the contrast could arise from small differences in shading, view factor or construction rather than from the finish alone.

### 3.3. Roof Radiation-Sensor Response

**Figure 7:**
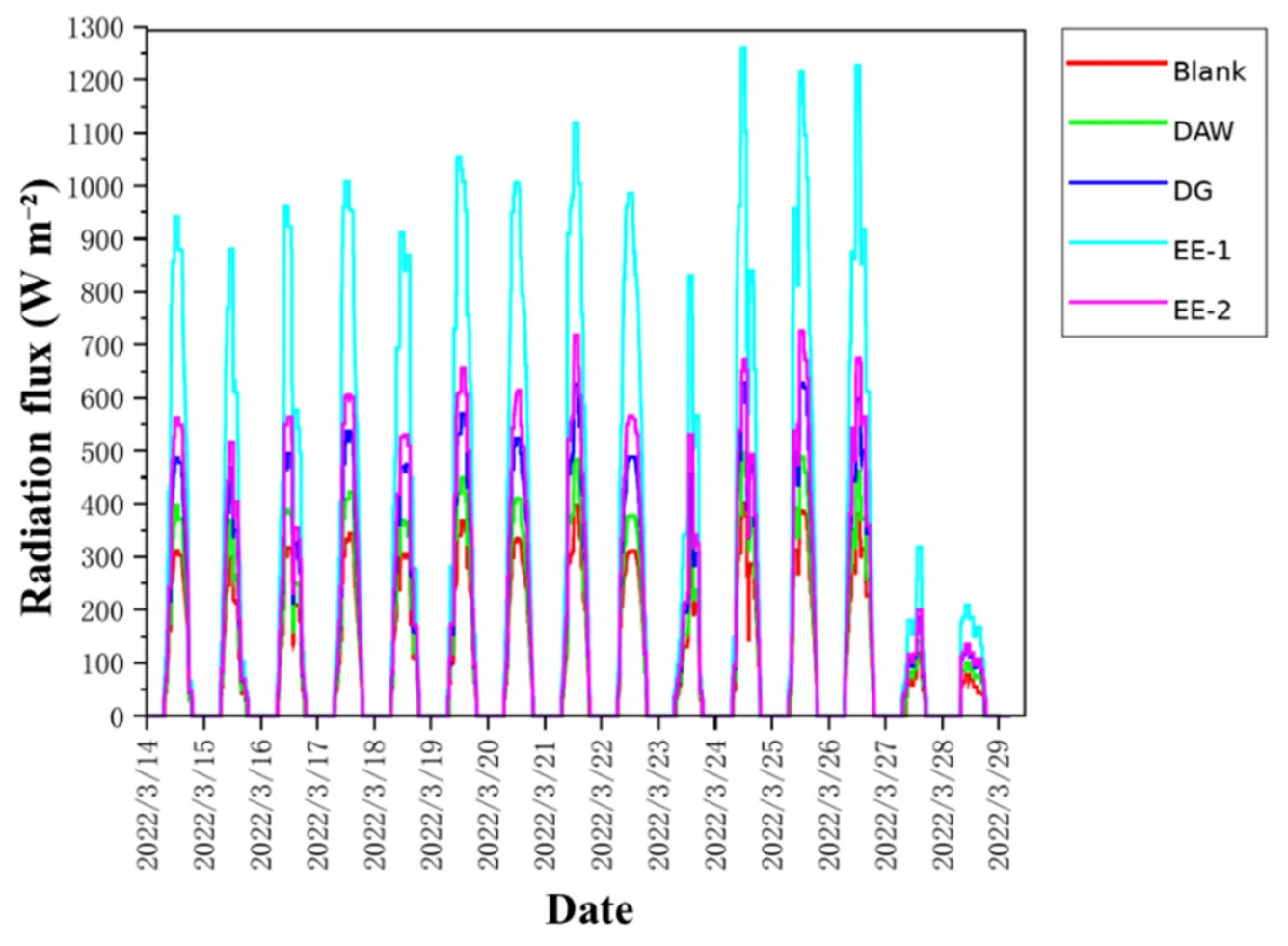
Radiation flux recorded at the roof centre of each house by broadband sensors (W m^−2^).

A high-reflectance roof should reject a larger fraction of incident radiation than a high-emittance control. Roof-centre broadband reverse-radiation sensors were therefore used only as a comparative indicator. They ranked EE-1 highest, with a peak of approximately 1300 W m^−2^; EE-2 reached ~700 W m^−2^ (Figure 7). These peaks lie in the same range as concurrent solar irradiance (700–1300 W m^−2^) and far above typical downward atmospheric long-wave fluxes of a few hundred W m^−2^. They are reported as a comparative measure of rejected incident radiative energy, expected to be dominated by reflected short-wave radiation, not as calibrated downward long-wave irradiance and not as evidence of enhanced sky-window cooling. Spectral response and a full calibration certificate for these sensors were not archived beyond the campaign description. A paired pyranometer/pyrgeometer campaign would be required to separate reflected short-wave from emitted long-wave radiation and was not performed.

### 3.4. Air-Conditioning Electricity Use

**Figure 8:**
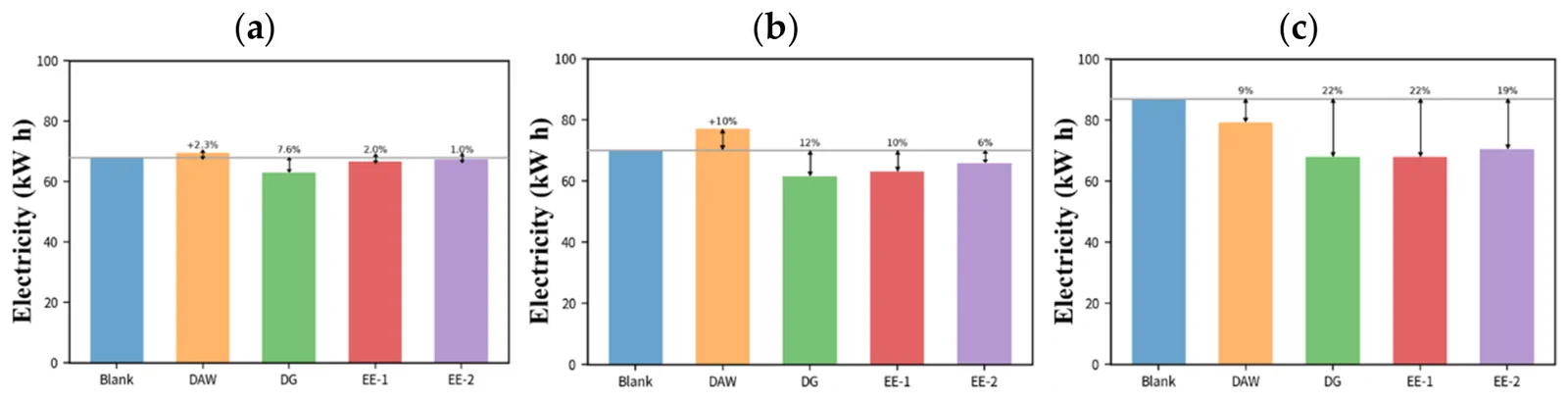
Air-conditioner electricity use at three indoor set-points: (**a**) 26 °C; (**b**) 23 °C; (**c**) 20 °C. Percentages are relative to the contemporaneous blank house. Bars below the reference line are savings; the DAW values +2.3% (26 °C) and +10% (23 °C) are increases in consumption.

For building applications, the decisive metric is not surface temperature alone but the electricity that air-conditioning must still consume. When the air-conditioners were operated at fixed set-points from 7 May to 24 June 2022 (no added interior insulation), relative savings versus the blank house were 2.0% (EE-1) and 1.0% (EE-2) at 26 °C; 10% and 6% at 23 °C; and 22% and 19% at 20 °C (Figure 8). Over the same windows, DG used 7.6%, 12% and 22% less electricity than the blank house. DAW used 2.3% more electricity than the blank house at 26 °C and 10% more at 23 °C (no saving in either window) and 9% less at 20 °C. In Figure 8, the DAW labels of 2.3% (26 °C) and 10% (23 °C) are consumption increases relative to the blank house; labels on bars below the blank-house reference line are savings. The 26 °C interval corresponds to a blank-house use of approximately 68–70 kW h on the original bar chart. The campaign report also tabulates monthly-normalized savings on a 4 m^2^ floor-area basis (kW h m^−2^ month^−1^): EE-1 16.3/3.88/0.76 and EE-2 14.0/2.28/−0.4 at 20/23/26 °C. The 1–2% EE contrasts at 26 °C, and the 2.3% DAW increase in the same window, are comparable to plausible logger uncertainty and are not used as primary evidence. After interior XPS insulation was added, a separate check on 26–30 August 2022 at 24 °C gave relative savings of 2.7% (EE-1) and 2.0% (EE-3); DG saved 4.2% and DAW none. In a further passive interval on 9–14 September 2022 (ambient maxima 34–36 °C), indoor air was about 29–30 °C (DAW), 30–31 °C (DG), 31–32 °C (EE-1) and 32–33 °C (EE-3). The insulated-house ranking is therefore weaker than the May–June uninsulated ranking, consistent with the lower solar reflectance (0.72) and higher comparative emissivity (0.52) of EE-3. Absolute metre exports for every interval were not recovered from the logger archive.

### 3.5. Durability Under Tropical Weathering

**Figure 9:**
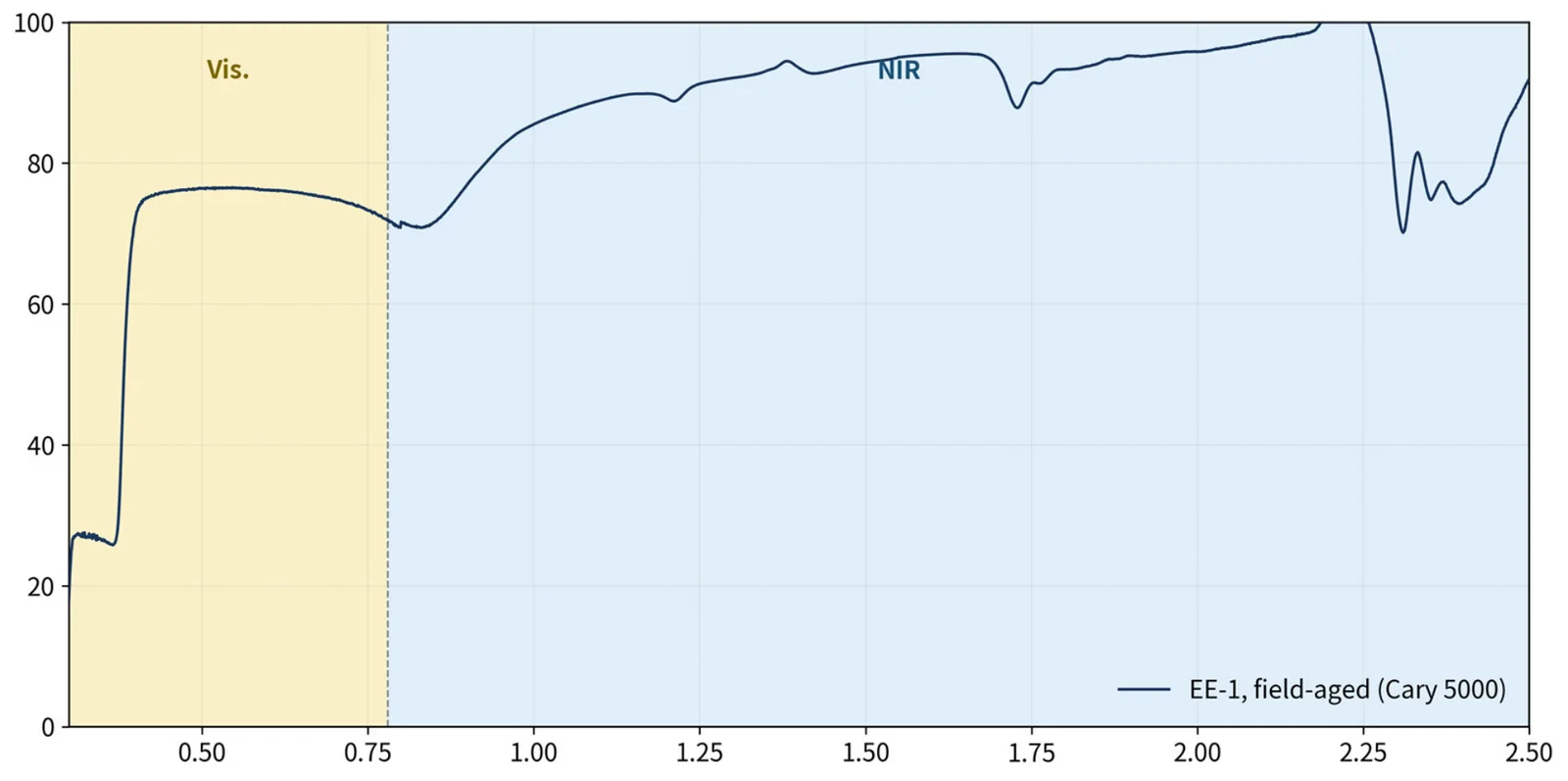
Spectral reflectance of field-aged EE-1 after outdoor exposure in Hainan (March–July 2022), recorded on an Agilent Cary 5000 spectrophotometer (Agilent Technologies, Santa Clara, CA, USA) (%R, λ = 0.25–2.50 µm). The yellow band is the visible range (0.30–0.78 µm) and the blue band is the near-infrared range (0.78–2.50 µm). Solar reflectance of the same coupon, determined according to GB/T 25261-2018, was 0.769.

**Figure 10:**
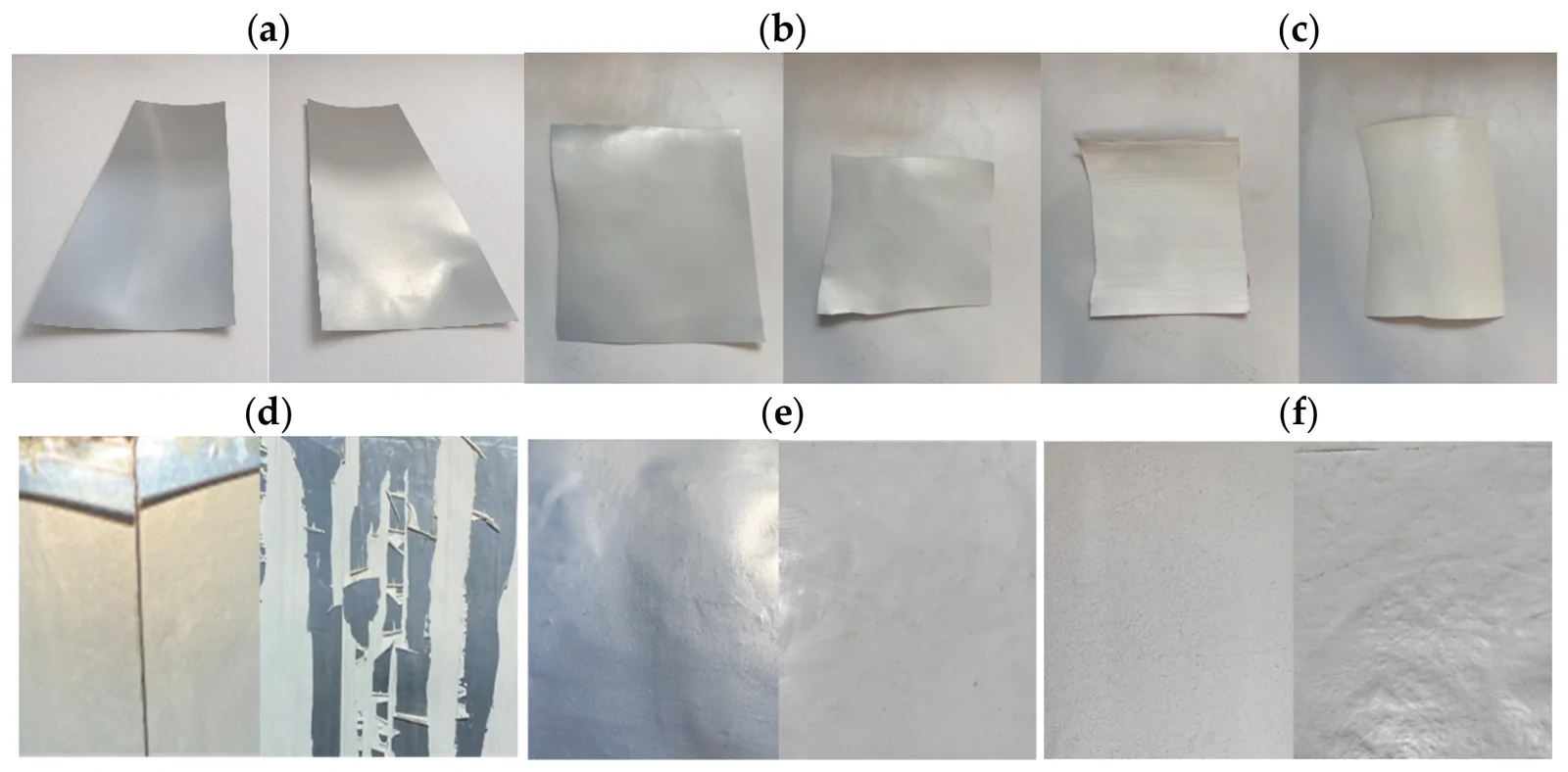
Appearance of the tested finishes before and after approximately four months of outdoor exposure in Hainan. (**a**) EE-1 and EE-2 before exposure; (**b**) EE-3 and EE-4 before exposure; (**c**) DAW and DG before exposure; (**d**) EE-1 and EE-2 after exposure; (**e**) EE-3 and EE-4 after exposure; (**f**) DAW and DG after exposure.

Long-term durability under combined ultraviolet radiation, high humidity and thermal cycling is a prerequisite for any exterior envelope finish. After nearly four months outdoors (March–July 2022), EE-1 showed interfacial delamination (Al/PVC and PE/Al), and EE-2 showed severe PE-surface damage, consistent with 720 h laboratory weathering and with the weak PE/Al and Al/PVC peel of EE-1. EE-3 and EE-4 showed no obvious visual change over a similarly short field period (Figure 10), in line with the laboratory finding that the EE-3 PE/Al interface could not be peeled. Appearance checks remain preliminary for year-scale service. For EE-1, post-exposure solar reflectance, hemispherical emissivity (Figure 9) and dumbbell tensile strength (GB/T 528-1998) were measured on retrieved coupons and are discussed above. Interlayer peel after exposure was not remeasured because the retrieved coupons were insufficient for GB/T 8808-1988; tensile strength and elongation are used instead as the mechanical record. Post-exposure optical values for EE-3/EE-4 are not reported, for the house-assignment reason given in the Discussion. Year-scale outdoor exposure remains to be reported.

Figure 2 compares an as-produced EE-1 cross-section with a coupon taken after the same tropical exposure that produced visible delamination. In the unexposed sheet (Figure 2a), a particle-filled ply consistent with the PVC core lies against a thinner, more compact polymer skin; the interface between these plies is already distinct. After exposure (Figure 2b), MPa (TD) (*n* = 5 each), i.e., 103% and 105% of the unexposed campaign values (26.5 MPa MD, 26.92 MPa TD). The small apparent increase lies within ordinary coupon-to-coupon scatter and is not treated as strengthening. Mean elongation at break was 70.45% (MD; range 24.13–140%) and 42.88% (TD; range 27.69–53.07%), i.e., 86% and 58% of the unexposed values (81.56% MD, 74.21% TD). Tensile strength at break was lower and more anisotropic (12.15 MPa MD, 17.45 MPa TD), as expected once plies have separated and the peak load is not sustained to rupture. Peak tensile strength of EE-1 was therefore retained after the Hainan exposure; ductility, especially in the transverse direction, was not. Interlayer peel after exposure was not remeasured because the retrieved area was insufficient for GB/T 8808-1988. The tensile change is the available mechanical record. It cannot be read as a peel strength, and the missing post-exposure peel test leaves the adhesion loss unquantified.

## 4. Discussion

The measured comparative 8–13 µm emissivity of 0.32–0.38 is the opposite of an ideal passive-daytime-radiative-cooling emitter [6,7,8,9,10,11]. Combined with solar reflectance of ~0.77, the stack is a solar reflector plus radiant barrier. The experimentally supported benefit is the reduction in absorbed solar load: exterior-roof temperature fell by up to 8 °C on EE-1 relative to the contemporaneous blank house in May. Low emissivity is a second, inferred contribution. By Kirchhoff’s law, it lowers long-wave absorptance, but it also lowers emission from the exterior surface, so the net long-wave term depends on whether the surroundings are hotter or colder than the wall. That split was not measured. The roof-centre peaks near 1300 W m^−2^ lie in the range of solar irradiance and are treated only as a comparative indication of rejected incident radiation. The campaign 0.32–0.38 values are comparative estimates from ε = 1 − ρ; the post-exposure value 0.34 is a laboratory hemispherical emissivity and is not the same measurand. Indoor air did not fall below ambient without air-conditioning, so sub-ambient cooling is not claimed.

A simplified exterior-surface statement helps separate the two optical contributions. Absorbed solar radiation αG, absorbed long-wave radiation εL↓, convection and conduction into the 120 mm wall are balanced by emitted long-wave radiation εσTs^4, with α = 1 − ρ_solar taken from the laboratory spectra and G from the JLC-QTS station. Near noon, the reduction in αG between the blank roof and EE-1 is several hundred W m^−2^ and is the first-order cause of the lower exterior temperature. Lowering ε from ~0.85 to ~0.35 changes both absorbed and emitted long-wave fluxes; in hot surroundings the net long-wave change is a radiant-barrier reduction in gain, smaller than the solar term at midday but relevant on walls and interiors. The convective coefficient was not measured on site; a literature range consistent with 2–7 m s^−1^ is assumed. The decomposition is therefore an order-of-magnitude estimate, not an experimentally separated two-term measurement. May temperature reductions (8 °C exterior roof, 5 °C interior roof, 4 °C indoor air versus the blank house) are consistent with that picture. They should not be read as proof that the five houses were thermally identical before coating.

If the wallpaper lowers the heat flux entering the envelope, relative electricity savings can grow when the indoor–outdoor temperature difference increases. Relative savings did increase as the set-point was lowered from 26 to 20 °C. A linear fit of savings versus set-point gave R^2^ > 0.97, but only three set-points were tested, and the relation is indicative, not a physical law. The 1–2% EE contrast at 26 °C and the 2.3% DAW increase at the same set-point are not used as primary evidence. DAW showed no savings at 26 °C or 23 °C and a 9% saving only at 20 °C. Magnitudes at 23 and 20 °C are consistent in direction with 10–30% cool-roof savings reported elsewhere [4,12,13], but the present tests used small unoccupied boxes over days to two weeks and must not be read as annual whole-building savings. A cost advantage of the EE wallpaper cannot be claimed from the campaign report. The figure of ¥241 m^−2^ tabulated there is the installed cost of the DG comparator only (¥91 m^−2^ sheet plus ¥150 m^−2^ tape). The EE sheet price is listed as undisclosed (“—”). EE-1 and EE-2 used the same class of tape (¥150 m^−2^ in that table), so even the installation-adhesive item is not cheaper than DG. DAW coating is listed at ¥16.1 m^−2^ installed. Without a released EE factory price, no ranking of EE versus DAW or DG on material cost, and no payback period, is justified.

Durability is the main limit on practical use. EE-1 delaminated at metal–polymer interfaces, and EE-2 showed severe PE-surface damage after 3–4 months, so the formulations that carried the May–June temperature and electricity results did not remain intact. EE-3 was installed on house 5# only after EE-2 had failed. It was used for a short insulated check at 24 °C (2.0% electricity difference) and a three-month appearance screen, not for the uninsulated May–June campaign. Retrieved coupons that can be traced to a house number belong to house 4# (EE-1). A later file labelled EE-4 could not be matched to that archive and is not reported. Improved visual stability and the reported cooling are therefore not properties of one formulation. On EE-1, post-exposure solar reflectance was 0.769 and hemispherical emissivity was 0.34. Peak tensile strength was retained (27.18 MPa machine direction, 28.14 MPa transverse; 103% and 105% of the unexposed sheet) while transverse elongation fell to 58%. These tensile changes show that the sheet could still carry a peak load; they do not measure interlayer adhesion. Peel after exposure was not remeasured because the retrieved area was insufficient for GB/T 8808-1988. Water-vapour permeability, moisture transport and substrate-adhesion durability were not measured. The aluminized ply is expected to impede vapour transport, which is relevant for both exterior and interior use and remains unquantified. Year-scale outdoor exposure has not been reported. On the present evidence, the wallpaper is a candidate finish for short instrumented trials, not a demonstrated multi-season envelope product.

The houses are ~2.1 m cubes, unoccupied and free of realistic internal gains. One house was used for each finish. Treatments were not randomized, and no pre-treatment uncoated baseline exists, so temperature and electricity differences cannot be attributed solely to the surface. Spacing of 1.8 m reduces but does not remove neighbour and ground long-wave effects. Set-point windows covered different dates and weather, so percentage savings are not a single ranking. The roof sensors are broadband devices, not calibrated pyrgeometers. Figure 2 shows ply-scale separation but does not locate which metal–polymer interface opened first. DG saved 7.6%, 12% and 22% relative to the blank house at 26, 23 and 20 °C, comparable to EE-1 at the colder set-point. The EE sheet price was not disclosed, so no cost or payback comparison with DG or DAW is made.

## 5. Conclusions

A tape-applied four-layer wallpaper with a commercial aluminized interlayer provides solar reflectance of ~0.77 (EE-1/EE-2; 0.72 for EE-3), near-infrared reflectance of 0.74–0.81 and comparative 8–13 µm emissivity of 0.32–0.38 (0.52 for EE-3). On uninsulated Hainan model houses, with one house per finish and no pre-treatment baseline, EE-1 showed exterior-roof and indoor-air temperature contrasts of up to 8 °C and about 4 °C versus a contemporaneous uncoated house, and electricity contrasts of 10% and 22% at 23 and 20 °C. The 2% contrast at 26 °C is within likely monitoring uncertainty. These contrasts are descriptive. The demonstrated contribution is solar reflection; the net effect of low emissivity was not separated experimentally. After 3–4 months, EE-1 delaminated, and EE-2 suffered PE-surface damage. Retrieved EE-1 retained solar reflectance (0.769) and peak tensile strength, with transverse elongation reduced to 58% of the unexposed value; post-exposure peel was not measured. EE-3/EE-4 improved short-term appearance only. Cooling effectiveness and multi-month durability were not demonstrated on the same formulation. Water-vapour permeability and long-term substrate adhesion were not measured, and no cost advantage over DG is claimed. The sheet is not presented as a durable substitute for the commercial references.

## Data Availability

The original contributions presented in this study are included in the article. Further inquiries can be directed to the corresponding author.

## Abbreviations

The following abbreviations are used in this manuscript:

FTIR	Fourier-transform infrared spectroscopy
HVAC	Heating, ventilation and air-conditioning
NIR	Near-infrared
PDRC	Passive daytime radiative cooling
PE	Polyethylene
PET	Polyethylene terephthalate
PVC	Polyvinyl chloride
UV	Ultraviolet

## References

[1] Pérez-Lombard L., Ortiz J., Pout C. (2008). A review on buildings energy consumption information. Energy Build..

[2] González-Torres M., Pérez-Lombard L., Coronel J.F., Rodríguez-Maestre I., Yan D. (2022). A review on buildings energy information: Trends, end-uses, fuels and drivers. Energy Rep..

[3] Shukla A.K., Yadav A.K., Prakash R. (2023). Active and passive methods for cooling load reduction in a tropical hostel building. Energy Convers. Manag..

[4] Synnefa A., Santamouris M., Akbari H. (2007). Estimating the effect of using cool coatings on energy loads and thermal comfort in residential buildings in various climatic conditions. Energy Build..

[5] Zhao B., Hu M., Ao X., Chen N., Pei G. (2019). Radiative cooling: A review of fundamentals, materials, applications, and prospects. Appl. Energy.

[6] Raman A.P., Anoma M.A., Zhu L., Rephaeli E., Fan S. (2014). Passive radiative cooling below ambient air temperature under direct sunlight. Nature.

[7] Yin X., Yang R., Tan G., Fan S. (2020). Terrestrial radiative cooling: Using the cold universe as a renewable and sustainable energy source. Science.

[8] Li W., Fan S. (2019). Radiative cooling: Harvesting the coldness of the universe. Opt. Photonics News.

[9] Zhai Y., Ma Y., David S.N., Zhao D., Lou R., Tan G., Yang R., Yin X. (2017). Scalable-manufactured randomized glass-polymer hybrid metamaterial for daytime radiative cooling. Science.

[10] Mandal J., Fu Y., Overvig A.C., Jia M., Sun K., Shi N.N., Zhou H., Xiao X., Yu N., Yang Y. (2018). Hierarchically porous polymer coatings for highly efficient passive daytime radiative cooling. Science.

[11] Zeng S., Pian S., Su M., Wang Z., Wu M., Liu X., Chen M., Xiang Y., Wu J., Zhang M. (2021). Hierarchical-morphology metafabric for scalable passive daytime radiative cooling. Science.

[12] Levinson R., Akbari H. (2010). Potential benefits of cool roofs on commercial buildings: Conserving energy, saving money, and reducing emission of greenhouse gases and air pollutants. Energy Effic..

[13] Akbari H., Levinson R., Rainer L. (2005). Monitoring the energy-use effects of cool roofs on California commercial buildings. Energy Build..

[14] Qi G., Tan X., Yang X., Qiao Y., Li X., Wang Y., Chen S., Tu Y., Nie S., Yan K. (2024). Anti-aging and flexible-porous-array films for radiative cooling. Sol. Energy Mater. Sol. Cells.

[15] Wu J.Y., Xia L.M., Wu J.K., Yang Y.Y., Zhong W.H., Li J.G., Yu Y., Yang R.L. (2026). Self-adaptive non-flammable wallpaper with layered bead-network structure for light path modulation enabling on-demand building thermal management. Adv. Sci..

[16] (2001). Plastics Film and Sheeting—Determination of Thickness by Mechanical Scanning.

[17] (2001). Plastics Film and Sheeting—Determination of Length and Width.

[18] (2012). Polymer Water-Proof Materials—Part 1: Water-Proof Sheet.

[19] (1988). Test Method for Peel Force of Flexible Laminated Plastics.

[20] (2000). Test Methods for Weathering Resistance of Building Waterproofing Sheets.

[21] (2008). Test Method for Artificial Accelerated Weathering of Building Materials.

[22] Flexible Cellular Polymeric Materials—Determination of Low-Temperature Bending.

[23] (2014). Synthetic Resin Emulsion Coatings for Exterior Wall.

[24] (2018). Solar Heat Reflecting Insulation Coatings for Buildings.

[25] (1998). Rubber, Vulcanized or Thermoplastic—Determination of Tensile Stress-Strain Properties.

